# Anti-acetylcholinesterase and Antioxidant Activity of Essential Oils from *Hedychium gardnerianum* Sheppard ex Ker-Gawl 

**DOI:** 10.3390/molecules17033082

**Published:** 2012-03-12

**Authors:** Miguel Arruda, Hugo Viana, Nuno Rainha, Nuno R. Neng, José Silvino Rosa, José M. F. Nogueira, Maria do Carmo Barreto

**Affiliations:** 1 Department of Technological Sciences and Development (DCTD), University of Azores, Rua Mãe de Deust, 9501-801 Ponta Delgada, Portugal; 2 Centro de Investigação de Recursos Naturais (CIRN), University of Azores, 9501-801 Ponta Delgada, Portugal; 3 Department of Biology, Centro de Investigação em Biodiversidade e Recursos Genéticos (CIBIO-Azores), University of Azores, Rua Mãe de Deus, 9501-801 Ponta Delgada, Portugal; 4 Faculty of Sciences, Chemistry and Biochemistry Department and Center of Chemistry and Biochemistry, University of Lisbon, Campo Grande Ed. C8, 1749-016 Lisbon, Portugal

**Keywords:** *Hedychium gardnerianum*, essential oils, anti-acetylcholinesterasic activity, antioxidant activity, Alzheimer’s disease

## Abstract

Acetylcholinesterase inhibition, antioxidant and cytotoxic activities of *Hedychium gardnerianum* leaf essential oils from S. Miguel Island were determined. All the oils inhibited acetylcholinesterase, with IC_50_ values of approximately 1 mg/mL, showing no statistical differences between collection sites. Three oils presented mixed inhibition, whilst one was almost truly competitive. This activity can be attributed to the presence of sesquiterpenes, which constituted more than 60% of the composition of the oils. Regarding the antioxidant activity as measured by the DPPH method, all the oils presented activities similar to reference compounds, although with statistical differences between collection sites. Cytotoxicity measured using *Artemia salina* classified these oils as moderately toxic, with LC_50_ values ranging from 300 to 500 µg/mL. These results indicate a possible application of these oils in aromatherapy as coadjuvants in the treatment of cognitive diseases such as Alzheimer, since they may contribute to increase acetylcholine in cholinergic neurons and simultaneously fight deleterious oxidations responsible by neurological degeneration.

## 1. Introduction

Numerous plant extracts are used for therapeutic purposes because they contain several active substances, some of which are used to treat diseases related to the Central Nervous System (CNS). Many are known to have beneficial effects on memory disorders, depression and cerebral ischemia [1,2]. The use of essential oils on the treatment of cognitive disorders has been done on an empirical base for many years, although some of those effects have been scientifically proved (e.g., [2,3]). 

Alzheimer’s disease (AD) is a neurodegenerative disorder that affects nearly 2% of the population in industrialized countries, and is characterized by the reduction of the brain regions involved in learning and memory processes, as the result of degeneration of synapses and death of neurons [4]. The majority of FDA-approved drugs for AD (e.g., tacrine, donepezil, rivastigmine, galantamine) act by countering the cholinergic deficit associated with cognitive dysfunction and are based on the inhibition of the enzyme acetylcholinesterase (AChE) [5,6]. However, although these drugs contribute to slow down the progress of the disease, they cause negative side effects (e.g., see [7]) therefore the search for sources of anti-acetylcholinesterasic compounds which are both effective, selective and with less negative effects is extremely important. Oxidative stress has also been associated with the progression of AD and other chronic conditions related to aging processes [8], which means that some mixtures of compounds with both anti-acetylcholinesterasic and antioxidant activity, may explain some of the effects of traditional herbal medicine, treating the whole disorder as opposed to single isolated symptoms of the disease [1].

*Hedychium gardnerianum* Sheppard ex Ker-Gawl. is a rhizomatous perennial herb of the Zingiberaceae family, named “conteira” in the Azores. It was introduced in the Azores from its native Himalayas in the middle of the 19th century, and is widespread on all of the Azorean islands, except Corvo. It also spreads rapidly wherever the native forest becomes degraded, and is also being scattered in the dense laurel forest of the island [9]. Recent studies have shown antithrombin and antibacterial activity of its essential oils [10] and cytotoxicity of labdane-type diterpenes isolated from this plant [11]. However, no work has been reported on its anti-acetylcholinesterasic and antioxidant activity. Bearing all this in mind, in this study the acetylcholinesterase inhibition properties, radical scavenging and cytotoxic activities are reported for *H. gardnerianum* leaf essential oils from four different locations of São Miguel Island (Azores). Gas chromatography with mass spectrometry detection (GC-MS) was used to determine the chemical composition of the oils. 

## 2. Results and Discussion

### 2.1. Essential Oil Composition

The essential oils presented average yields of 0.03%. The main components of the essential oils (Table 1) were sesquiterpene hydrocarbons (47.8 to 52.7%) and oxygenated sesquiterpenes (15.2 to 16.3%). Monoterpene hydrocarbons constituted less than 2%, which is different from the *H. gardnerianum* leaf oils extracted by Medeiros *et al*. [10], who reported approximately 15–30% of pinenes, although the values for sesquiterpenes were comparable to the present work. However, it is a known fact that the relative composition of components in the essential oils of a given source often varies remarkably with geographical location, season and the climate of a given year, beside other factors (e.g., [12,13]). 

**Table 1:** Composition (%) of the essential oils from *H. gardnerianum* mature leaves obtained by GC-MS.

Components				*%^b^*		
	Class	RI^a^	*FU*	*FO*	*SC*	*AC*
α-Pinene	a	870	0.9	0.8	0.8	0.8
β-Pinene	a	920	0.8	0.7	0.7	0.7
β-Limonene	a	986	0.1	t	0.1	0.1
Eucalyptol	b	996	n.d.	n.d.	t	t
S-3-Carene	a	1080	t	n.d.	t	t
*trans*-Limonene oxide	b	1128	0.1	0.1	t	0.1
Cymol	f	1136	t	0.1	0.1	t
*m*-Cymene	a	1178	t	n.d.	0.1	t
Verbenene	a	1195	0.1	n.d.	t	0.1
Myrtenal	b	1201	0.1	0.1	0.1	0.1
α-Copaene	c	1416	0.7	0.1	0.1	0.1
β-Elemene	c	1434	0.1	n.d.	0.1	n.d
*trans*-Caryophyllene	c	1465	0.5	0.4	0.6	0.4
α-Humulene	c	1501	2.7	2.2	3.3	2.1
Neoalloocimene	c	1510	1.1	1.1	1.2	1.3
(−)-Calamenene	c	1523	0.1	0.1	0.1	0.1
β-Selinene	c	1536	n.d.	n.d.	0.1	0.1
α-Selinene	c	1545	0.8	1.4	1.4	0.8
α-Muurolene	c	1551	1.1	0.9	1.0	0.9
α-Farnesene	c	1559	1.1	0.5	0.9	0.5
γ-Cadinene	c	1565	3.5	3.9	3.7	3.4
Cadalin	c	1576	6.4	5.0	5.9	5.0
α-Cadinene	c	1590	0.4	0.4	0.4	0.4
3-Methoxycinnamaldehyde	f	1620	n.d.	0.1	0.4	0.3
9,10-Dehydroisolongifolene	c	1632	4.8	5.6	5.0	4.8
8,9-Dehydroisolongifolene	c	1637	n.d	3.9	3.6	4.8
trans-α-Bisabolene	c	1653	4.2	4.2	4.5	4.0
3,4-Dimethyl-3-cyclohexen-1-carboxaldehyde	f	1664	9.0	10.2	9.0	10.5
1,2,3,4,4a,7-Hexahydro-1,6-dimethyl-4-(1-methylethyl)-naphthalene	c	1669	2.2	2.5	2.1	2.3
(−)-δ-Selinene	c	1672	0.7	0.4	n.d.	n.d.
Calamene	c	1680	n.d.	2.4	2.2	3.1
(−)-Cedreanol	d	1694	16.3	15.8	15.2	15.9
δ -Cadinene	c	1705	16.5	14.6	n.d.	n.d.
α-Calacorene	c	1711	1.1	1.1	15.9	16.2
Guaiazolene	c	1730	0.4	0.5	0.5	0.5
(−)-Isoledene	c	1741	0.3	0.3	0.3	0.3
11,14,17-Eicosatrienoic acid methyl ester	e	2116	0.1	n.d.	0.1	0.1
*Monoterpene hydrocarbons*	a		1.9	1.6	1.7	1.7
*Oxygenated monoterpenes*	b		0.2	0.1	0.2	0.2
*Sesquiterpenes hydrocarbons*	c		47.8	51.5	52.7	50.9
*Oxygenated sesquiterpenes*	d		16.3	15.8	15.2	15.9
*Esters*	e		0.1	n.d.	0.1	0.1
*Others*	f		9.0	10.4	9.5	10.9
Total			75.0	79.5	79.3	79.8

t – trace (<0.05%); *n.d.* – Not determined; ^a^ Retention index against C_7_-C_27_ *n*-alkanes; ^b^ Normalized peak abundances without correction factors. Place of sampling: FU = Furnas; FO = Fogo; SC = Sete Cidades; AC = Achada do Nordeste.

The oils of plants from the four sites were similar in the majority of the components, and the major compounds were (-)-cedreanol, 3,4-dimethyl-3-cyclohexen-1-carboxaldehyde, cadalin, δ-cadinene and α-calacorene. However, two groups, Furnas (FU) and Fogo (FO), and Sete Cidades (SC) and Achada (AC), can be formed based on the difference in α-calacorene and δ-cadinene levels. Essential oils from FU and FO were found to have a high level of δ-cadinene (approximately 15%) and low level of α-calacorene (1.1%), whereas oils from SC and AC were seen to contain α-calacorene and δ-cadinene above 15% and under the detection limit, respectively. 

### 2.2. Acetylcholinesterase (AChE) Inhibition Assay

The percent inhibition of AChE activity at the highest concentration tested, 2.5 mg/mL, was around 60% (between 59.8 and 62.4%), with no significant differences between the oils of plants from four sites (LSD test, P = 0.05).

The IC_50_ values obtained from the AChE inhibition assay for the essential oils are shown in Table 2. The strongest inhibition was displayed by FU samples, followed by FO and AC samples. SC samples exhibited an IC_50_ value of 1.37 mg/mL. The differences among the values for all samples were statistically insignificant. However, IC_50_ values were comparable to that of α-pinene, a known AChE inhibitor [14]. Thus, the samples studied in the present work can be considered as potential anti-acetylcholinesterasic sources, since extracts with IC_50_ value lower than 1.00 mg/mL were considered as strong anti-acetylcholinesterasic by many authors [7,15,16,17]. AChE inhibition can be explained by the high content in sesquiterpenes [18,19,20], although many other terpenes, such as monoterpenes and diterpenes, are also reported as strongly inhibiting the activity of this enzyme [19,21].

**Table 2:** Inhibition of AChE by essential oils from leaves of *H. gardnerianum* from four different sampling sites, and Standard compounds (α-pinene and ursolic acid).

Essential oils/standard	AChE inhibitory activity		
	IC_50_ (mg/mL)	K_M_ (mg/mL)	V_max_ (ΔAbs_415nm_/min)
**Control**	-	0.466	0.0587
**FU**	1.03 ^a^ ± 0.14	2.605	0.0689
**FO**	1.17 ^a^ ± 0.07	1.061	0.0390
**AC**	1.17 ^a^ ± 0.09	1.261	0.0312
**SC**	1.37 ^a^ ± 0.27	1.01	0.0389
**α-Pinene**	1.43 ^a^ ± 0.07	0.453	0.0477
**Ursolic acid**	0.19 ^b^ ± 0.014	2.410	0.0579

Place of sampling: FU = Furnas; FO = Fogo; SC = Sete Cidades; AC = Achada do Nordeste. Results are means ± standard deviation. Means marked by the same letter indicate no significant differences between essential oils *P* = 0.05 (LSD test). Michaelis constant (K_M_) and maximum velocity (V_max_) were determined for control without inhibitor, or in the presence of 1.25 mg/mL essential oil or compound.

### 2.3. Characterization of the Inhibition of Acetylcholinesterase

The inhibition type of the oils was analyzed by Lineweaver-Burk plots, yielding the values of *K_M_* and *Vmax* in the presence and absence of AChE inhibitor (Table 2). Compared to the control, the *Vmax* value was reduced in the presence of FO, FU and SC essential oils whilst the value of *Km* increased, indicating a mixed inhibition by these oils. Regarding the essential oil from AC, this was close to a competitive inhibition, since it had a higher *Km* value for the presence of the same oil, when compared with the control, and *Vmax* displayed roughly the same value. The fact that mixed inhibition was prevalent is mainly due to the complexity of essential oils, therefore the analysis of inhibition type reflects the sum of all the interactions between diverse compounds. This result is, however, an indication of the global effect of the essential oils, and therefore a useful tool in their use in therapeutics, and may contribute to clarify the interactions with the enzyme molecule.

### 2.4. DPPH Radical Scavenging Assay

The EC_50_ values for the DPPH radical scavenging potentials of the essential oils are shown in Table 3. All the essential oils exhibited high antioxidant activity to different extents, with EC_50_ values ranging between 8.5 and 31.1 μg/mL, which are potentially useful values when compared with the standard compounds quercetin, Trolox, ascorbic acid and BHT (3.1, 5.6, 10.3 and 31.0 μg/mL, respectively). Although the results were quite variable, no correlation was found with variations in the composition of the essential oils. Therefore, these variations may be due to variations in the composition of some of the compounds which are present in smaller concentrations, namely some of those present in the fraction which was not unambiguously identified. Antioxidant properties of essential oils often come from their monoterpene hydrocarbons, oxygenated monoterpenes and sesquiterpenes [20,22], which means that this strong antioxidant activity may be related to the sum of the effects of a complex mixture which had some variations between the four samples. Although it is generally considered that phenolic compounds are the main antioxidant molecules found in plant extracts, in essential oils the phenolic fraction is minor. Studies showed that essential oils may act by preventing lipid peroxidation, scavenging of free radicals and in very few cases, chelating metal ions. A recent review reports that the antioxidant activities of essential oils may act synergistically because their main components when used as references have less activity than the essential oil [23].

**Table 3:** Antioxidant activity of essential oils of *H. gardnerianum* collected at different locations.

Essential oils/standards	DPPH scavenging activity
	EC_50_ (µg/mL)
**FU**	8.46 ^a^ ± 0.90
**FO**	28.76 ^c^ ± 2.60
**AC**	31.14 ^c^ ± 2.70
**SC**	15.16 ^b^ ± 1.50
**Quercetin**	3.11 ± 0.06
**Trolox**	5.63 ± 0.09
**Ascorbic acid**	10.34 ± 0.28
**BHT**	31.00 ± 0.19

Place of sampling: FU = Furnas; FO = Fogo; SC = Sete Cidades; AC = Achada do Nordeste. Values are means ± standard deviations. Means marked by the same letter indicate no significant differences between essential oils *P* = 0.05 (LSD test).

### 2.5. Artemia salina Cytotoxicity Bioassay

The toxicity of the essential oils measured against *Artemia salina* is presented in Table 4. *A. salina* LC_50_ toxicity values were found to be within the range 300.1 and 504.7 μg/mL.

**Table 4:** LC_50_ concentrations of *H. gardnerianum* essential oils toxicity against *Artemia salina*.

Essential oils /Standard	A. salina Bioassay
	LC50 (μg/mL)
**FU**	375.93 a ± 24.91
**FO**	300.20 ab ± 15.80
**AC**	504.67 c ± 24.33
**SC**	379.93 b ± 94.90
**Berberine Chloride**	4.12 ± 0.02
**Phenol**	124.45 ± 0.23
**Caffeine**	>1,000
**Theophylline**	>1,000

Values of expressed in number of µg/mL of essential oil needed to kill 50% brine shrimp. LC_50_ > 1000 μg/mL are considered non-toxic. The values of LC in each column followed by the same letter are not significantly different. Place of sampling: FU = Furnas; FO = Fogo; SC = Sete Cidades; AC = Achada do Nordeste.

Thus, it can be concluded that these oils present moderate toxicity when compared to standard compounds such as berberine, which presents an LC_50_ of 4.12, and phenol, with an LC_50_ of 124.45 μg/mL. Compounds with LC_50_ values higher than or equal to 1,000 μg/mL were reported as non-toxic for *Artemia salina* [24].

## 3. Experimental

### 3.1. Chemicals

Acetylcholinesterase from *Electrophorus electricus* (electric eel) Type VI-S, ascorbic acid, butylated hydroxytoluene (BHT), α-pinene, Trolox, quercetin, 2,2-diphenyl-1-picrylhydrazyl and 5,5′-dithiobis-2-nitrobenzoic acid (DTNB) were acquired from Sigma-Aldrich^®^ Chemical Company (St. Louis, MO, USA). Methanol, analytical grade was obtained from Scharlab S.L. (Sentmenat, Spain). Sodium dihydrogen phosphate dihydrate and sodium chloride were bought on Merck® (Darmstad, Germany). Finally, disodium hydrogen phosphate and acetylcholine iodide (ATCI) were acquired from Fluka Chemicals^®^ (Buchs, Switzerland).

### 3.2. Plant Collection

The vegetable material was collected in São Miguel island and unambiguously indentified by an expert botanist (Doctor Luís Silva, from the Biology Department of the Azores University). *H. gardnerianum* mature leaves collected on four different sites in S. Miguel island: Furnas (FU) (37° 45’ 59,02”N and 25° 20’ 05,75”W), Fogo (FO) (37° 47’ 06,62”N and 25° 28’ 35,62”W), Sete Cidades (SC) (37° 50’ 44,69”N and 25° 45’ 46,12”W) and Achada do Nordeste (AC) (37° 49’ 39,28”N and 25° 15’ 28,35”W). The plant material was deposited in the herbarium of the Department of Biology of the University of Azores where voucher specimens are kept. 

### 3.3. Essential Oils Isolation

The essential oils from each sample of *H. gardnerianum* (2 kg) were obtained by hydrodistillation for 3 h in a modified Clevenger type apparatus. These were collected in a lighter than water oil graduated trap and stored at −20 °C until analyzed.

### 3.4. Essential Oils Analysis

The *H. gardnerianum* essential oils were analyzed on an Agilent 6890 gas chromatograph interfaced to an Agilent 5973N mass selective detector (Agilent Technologies, Palo Alto, CA, USA). A vaporization injector operating in the split mode (1:50) at 250 °C was used, into which a fused silica capillary column (30 m length × 0.32 mm internal diameter × 0.25 µm film thickness_; _ HP-5MS; 5% diphenyl 95% dimethyl polydimethylsiloxane; Agilent Technologies) was installed. The oven temperature was programmed at 60 °C (4 min) raised to 240 °C at 5 °C.min^−1^ and maintained at 250 °C for 5 min. Helium was used as carrier gas at 30 cm.s^−1^ and the injection volume was 1 µL. The transfer line, ion source and quadrupole analyzer temperatures were maintained at 280 °C, 230 °C and 150 °C, respectively, and a turbo molecular pump (10^−5^ torr) was used. In the full-scan mode, electron ionization mass spectra in the range 40–400 Da were recorded at 70 eV electron energy. A solvent delay of 4 min was selected. The acquisition data and instrument control were performed by the MSD ChemStation software (G1701CA; version C.00.00; Agilent Technologies). The identity of each compound was assigned by comparison of their retention index, relative to a standard mixture of n-alkanes [25], as well as by comparison with the mass spectra characteristic features obtained with the Wiley’s library spectral data bank (G1035B; Rev D.02.00; Agilent Technologies). For semi-quantification purposes the normalized peak area abundances without correction factors were used.

### 3.5. Microplate Assay for AChE Activity

The assay to measure AChE activity was modified from the assay described by Ellman *et al.* [26] and Ingkaninan *et al.* [15]. Briefly, 3 mM 5,5′-dithiobis[2-nitrobenzoic acid] (DTNB, 5 µL), 75 mM acetylthiocholine iodide (ATCI, 5µL), and sodium phosphate buffer 0.1 M (pH 8.0, 110 µL), and sample dissolved in buffer containing not more than 10% methanol (120 µL) were added to the wells followed by 0.25 U/mL AChE (10 µL). The microplate was then read at 415 nm every 2.5 min for 12.5 min in a Bio Rad Model 680 Microplate Reader (Bio-Rad Laboratories, Inc., Hercules, CA, USA). Percentage of enzymatic inhibition was calculated relatively to the control without inhibitor. Every experiment was done in triplicate. To determine IC_50_ values, EPA PROBIT ANALYSIS PROGRAM Version 1.4 was used. Assays to determine type of inhibition were performed in a similar manner, in sodium phosphate buffer 0.1 M (pH 8.0) with 10 µL of 3 mM DTNB, 240 μL of final volume and concentrations of ATCI between zero and 3mM in the presence or absence of test samples. Kinetic parameters K_M_ and V_max _ were calculated using double-reciprocal Lineweaver-Burke plots using Hyper 32 Version 1.0.0.

### 3.6. DPPH Radical Scavenging Assay

Antioxidant activity was assayed by the DPPH radical scavenging assay [27]. Serial dilutions of essential oils or reference compounds (trolox, BHT, quercetin and ascorbic acid) were carried out in 96-well microplates, to a methanolic DPPH solution at different concentrations and the absorbance at 517 nm was measured after 30 min in the dark. In each assay, a control was prepared, in which the sample or standard was substituted by the same amount of solvent. Percentage of antioxidant activity (%AA) was calculated as:

% AA =100 [1 − (A_control_ − A_sample_)/A_control_]

where A_control_ is the absorbance of the control and A_sample_ is the absorbance of the extract or standard. All assays were carried out in triplicate and results expressed as EC_50_.

### 3.7. Microwell Cytotoxicity Assay using Artemia salina (Brine Shrimp)

Cytotoxicity assays using *Artemia salina* were carried out as described by Solis *et al.* [24]. Samples for testing were dissolved in methanol (50 µL) and were made up to 1 mg/mL in artificial sea water. Serial dilutions were made in 96-well microplates in 100 µL artificial sea water. A suspension of nauplii containing 10–15 organisms (100 µL) was added to each well and the covered plate incubated at 25 °C for 24 h. Plates were then examined under a binocular microscope (×12.5) and the number of dead (non-motile) nauplii in each well were counted. Methanol (100 µL) was then added to each well and after 15 min the total numbers of shrimp in each well were counted and LC_50_ values calculated by Probit analysis. All experiments were done in triplicate and control wells with methanol were included in each experiment.

### 3.8. Statistical Analysis

Statistical analysis was carried out using SPSS 17.0. M.S. Data obtained was subjected to an arccosine transformation before proceeding to an analysis of variance (ANOVA). When the analysis was significant, means were separated by an LSD test, *P* < 0.05.

## 4. Conclusions

The essential oils of *H. gardnerianum* present both anti-acetylcholinesterasic and antioxidant activities to an extent which renders them potentially useful as an adjuvant in the treatment of cognitive diseases such as Alzheimer’s disease. The activities reported in the present work indicate that they may contribute to increase the levels of acetylcholine in cholinergic neurons, while simultaneously helping to prevent further degradations caused by radical oxygen species. Although the inhibition of acetylcholinesterase is lower than the activity of currently used active principles, which are mainly alkaloids, the essential oils have the advantage of being volatile and therefore may be used in aromatherapy, avoiding the secondary effects which are mainly connected with the digestive system. These volatile compounds also have the advantage of being able to readily cross the blood-brain barrier, due to their small molecular dimensions and lipophilicity. 
